# Impact of instructor on-slide presence in synchronous e-learning

**DOI:** 10.1007/s10639-022-11306-y

**Published:** 2022-09-09

**Authors:** Zoltan Katai, David Iclanzan

**Affiliations:** Faculty of Technical and Human Sciences Târgu-Mureş, Sapientia Hungarian University of Transylvania, Op. 9, Cp. 4, Târgu-Mureş, 540485 Romania

**Keywords:** Teaching/learning strategies, Distance education and online learning, Improving classroom teaching, Synchronous e-learning

## Abstract

As a consequence of the COVID-19 pandemic, many higher education programs had to switch to synchronous online teaching. Teachers suddenly faced pressing unaddressed challenges, such as how to better transfer their “presence” from the traditional classroom to the online space in a way that keeps students engaged. This paper explores new venues for increasing the quality of synchronous online learning. We propose the notion of broad on-slide presence, pillared on an increased instructor expressiveness and an elevated instructor slide-content interaction. We conducted four studies to investigate the benefits of delivering lectures in this format, using a mixed methods research approach. We combined survey methodology with transversal design and structural equation modelling with qualitative methodology using discourse analysis of teacher interviews. Results revealed a significant increase in perceived knowledge gain and attentional engagement, and an improved and more personal student experience. At the same time, the instructor’s broader on-slide presence also resulted in an increased teacher satisfaction.

## Introduction

Over the past decades, online course enrollments have grown substantially in higher education, reflecting the clear benefits of distance learning (Thomas et al., 2017; Allen & Seaman, 2016; Parsad et al., 2008). Besides this trend, the recent COVID-19 outbreak resulted in many universities moving their face-to-face studies online (Barratt & Duran, 2021). Because of the almost instant shift required, many students who opted for face-to-face learning were forced to accept distance education, where new topics are usually delivered through synchronous online lectures. In most countries, video conferencing tools provided a quick solution, enabling webinar-like courses. With slide supported lectures, the transition was straightforward as these meeting platforms have a screen sharing feature. Unfortunately, students often experienced the transition to be overall negative as the courses “became less enjoyable, less interesting, decreased in learning value, facilitated less attention and effort” (Garris & Fleck, 2020) and “inappropriate and inefficient, with few opportunities for interaction and discussion” (Duraku, 2021, p. 130). These circumstances and the importance of pandemic preparedness makes the research on the quality of synchronous online learning a priority.

Most previous research focused on the quality of online learning in asynchronous format (Hrastinski et al., 2010). However, it is quite a different challenge to deal with this issue in the context of synchronous online education. Quality video lectures are usually backed by a whole video production team and are often the result of significant postproduction (Madariaga et al., 2021). In contrast, quality synchronous lessons assume a “one-man live show” (especially during social distancing requirements), as the instructor usually implements these in real time by themselves, from their home or office. With the onset of the pandemic, companies quickly recognized the prevalence of this new use case and very recently started offering commercial solutions, such as the XSplit Presenter suite or the new Virtual Background feature in the Zoom videoconferencing platform. All these commercial solutions streamline for the average user the process of displaying content and presenters together, enabling more immersive presentation experiences.

A key notion in many studies on online learning is presence. For example, the Community of Inquiry (CoI) framework, which has become a prominent model of teaching and learning in online environments, is built on this concept. The originators of CoI (Garrison et al., 1999) initially defined three presences. Recently (Kozan & Caskurlu, 2018) published a review entitled *“On the Nth presence for the Community of Inquiry framework”* that extends the framework to seven presence dimensions. In the present study, we focus on the construct of instructor presence (IP) as it was conceptualized by (Richardson et al., 2015).

Crook & Schofield (2017) examined the concept of IP relative to traditional lectures. This approach aligns with the perspective of the Media Naturalness Theory (MNT) (Kock, 2005), which is another leading theory for examining online learning environments, especially the synchronous ones. According to MNT, effective online learning should aim to reach the highest degree of naturalness, similar to face-to-face interactions (Kock, 2005; Weiser et al., 2018). This perspective may be particularly justified when online education has been introduced as a substitute for face-to-face learning. We emphasize this point because there are also studies that conclude that students who chose online education did not attach particular importance to synchronous or face-to-face teacher-student communication (Beaudoin et al., 2009; Sheridan & Kelly, 2010; Mupinga et al., 2006).

Some previous research has suggested that including visuals of the instructor in video lectures has the potential to increase the impact of IP in online learning environments (Kizilcec et al., 2015; Wang et al., 2020). Presumably, this approach may have additional relevance in synchronous environments. Considering that over the last decade app-supported video chat has become an increasingly common mode of computer-mediated synchronous communication (Miller et al., 2017), it is conceivable that seeing their teacher would be an implicit expectation for many students who attend synchronous online lectures. On the other hand, there are studies that have revealed potential shortcomings when the teacher’s face and slide content are displayed in separate windows: i) attention division between the two video inputs (Schmidt-Weigand et al., 2010), ii) poor teacher-content communication (Friesen & Osguthorpe, 2018), and iii) limited instructor expressiveness. Weiser et al. (2018) highlighted a key limitation in that even modern video conferencing tools only partially convey the body language of the participants (Blau et al., 2017). A growing body of evidence from neuroscience and psychological studies suggests that limiting body language restricts instructors from expressing themselves “socially and pedagogically” (Calbi et al., 2017; Richardson et al., 2015).

Being triggered by the above limitations and aiming to address the clear research gap regarding slide-based synchronous online lectures, we developed a new, easy-to-integrate, platform-independent feature for video conferencing programs in the form of a client-side web application. The software processes the webcamera feed and projects only the instructor’s figure onto the slides in real time, generating a customizable virtual scene closer to what students are used to in in-person lectures. We approached the tool development from the perspective of MNT with the goal to get closer to the face-to-face learning format. On the other hand, the study we conducted focuses on the potential benefits of the new tool in increasing the impact of IP (CoI perspective), generating a so-called “broad on-slide presence” phenomenon. In addition, since we put a particular emphasis on instructor body language (faces belong to bodies), related research from the field of neuroscience provided a third perspective.

The main contribution of this study is to present a significant new development in the field of synchronous e-learning that it reflects on the improvement of using the existing slide-presence of instructors by enhancing their presence. We examined whether and where the generated learning experience improves students’ perception on the quality of the learning process. Our investigation also includes the qualitative analysis of four instructor interviews on the educational gains generated by the new tool.

## Background

As mentioned above, mainly the Community of Inquiry model, the Media Naturalness Theory, and recent neuroscience and psychological findings provided the theoretical framework for this research. We refer to these perspectives as complementary, since i) MNT explores the degree of naturalness of communication channels compared to face-to-face communication, ii) CoI explores the level of “three presences” that characterize the teaching and learning experience, and iii) the related neuroscience and psychological studies explore the opportunities and benefits of showing much more than a face.

### The CoI perspective

**Fig. 1:**
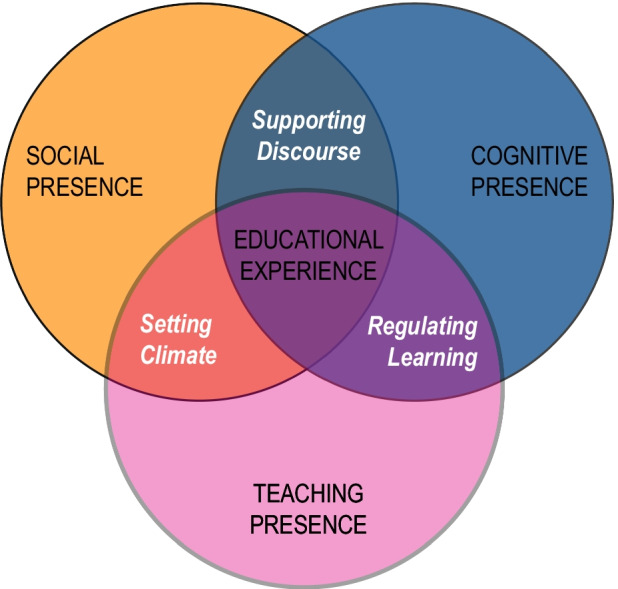
Community of Inquiry Framework

CoI was originally proposed by Garrison et al. (1999) and, over the past two decades, has become a prominent model of teaching and learning in online environments (Kozan and Caskurlu, 2018; Caskurlu et al., 2021). The CoI framework defines three presences (cognitive presence - CP, social presence - SI, and teaching presence - TP) and a large and increasing body of evidence has confirmed that each of these components contributes to student outcomes (e.g., satisfaction, perceived learning, actual learning, and retention) e.g., Richardson & Swan (2003), Richardson et al. (2017), Lim (2018), Caskurlu et al. (2020), and Turk et al. (2022). Figure 1 shows the iconic diagram of the CoI framework including the three presences and their areas of overlap: supporting discourse, setting climate and regulating learning.

From the perspective of TP, the primary role belongs to the teacher. On the other hand, the CP component focuses especially on students. Regarding SP, Thomas et al. (2017) noted that, although the originators of CoI emphasized that many of the online instructor responsibilities overlap with the construct of SP (Anderson et al., 2001), this component was mainly projected on students. To bridge this limitation, some authors distinguish between instructor social presence and the teaching presence component of CoI (Lowenthal, 2016). Swan & Shih (2005) reported a larger impact of instructor social presence on course outcomes than student social presence. Accordingly, we refer to IP as having two elements: instructor social presence and instructor teaching presence. Richardson et al. (2015) also conceptualized the IP construct as falling “at the intersection of teaching presence and social presence within the CoI framework”.

#### Increasing instructor social presence

SP was originally defined as “the degree of salience of the other person” (Short et al., 1976, p. 65) in mediated communication. These authors described SP as an objective attribute of the instrument based on the amount of communication cues it was able to convey. However, a transition has occurred over the years and the focus has shifted from the communication tool to the communication behavior of the participants (Thomas et al., 2017). Accordingly, Garrison et al. (1999) described SP as an “ability of participants in the Community of Inquiry” (p. 89).

This dual nature of the concept of SP (attribute of the tool vs. communication behavior of the participants) suggests two complementary approaches to increase the effect of this component: i) improving the instrument and ii) supporting participants in cultivating SP. By developing a tool that allows instructors to project their personal characteristics into the learning environment more powerfully and authentically, we have proposed to consider both aspects.

#### Increasing instructor teaching presence

According to Anderson et al. (2001), TP begins with designing an online course and continues throughout its implementation. This component of CoI has been conceptualized by three subdimensions: i) instructional design and organization (designing and planning course content, structure and process, student interactions, and assessment components); ii) facilitating discourse (sustaining “the interest, motivation and engagement of students in active learning”) (p. 7); iii) direct instruction (using “the subject matter and pedagogical expertise of the teacher” to support students in their own knowledge construction process) (p. 8).

Some studies conclude that the most important factors that influence online student satisfaction include content and organization, convenience and flexibility, online interaction, and instructor feedback (Beaudoin et al., 2009; Sheridan and Kelly, 2010). For example, Sheridan & Kelly (2010) found that the indicators of instructor presence that were most important to students were clear course requirements, responsiveness to student needs, timeliness of information, and instructor’s role, especially in providing feedback. In addition, these authors reported that their respondents rated the items “Provide a video that allows me to hear and see the instructor” and “Engages in real time chat sessions” as less important behaviors. On the other hand, other studies have come to different conclusions. For example, as Richardson et al. (2015) noted that students perceive the presence of the instructor primarily during the live part of the online course.

Since our study falls within the context of synchronous online education, we focused on increasing instructor’s teaching presence mainly along subdimensions ii) and iii). Subdimension i) is indirectly involved. Since the tool allows the instructor to change its location and size on the slide (to appear on or disappear from the slide, etc.), adequately designed slides leaves room for a more efficient exploitation of communication elements such as eye gaze, gestures, and body orientation, which, in turn, can result in more effective learning. Several studies (Pi et al., 2020; Cooney et al., 2017; Paulus et al., 2016) have provided evidence that learners can use the instructor’s eye gaze and body orientation to understand the focus of its attention.

### The MNT perspective

The MNT (Kock, 2005) focuses on the degree of medium naturalness by comparing its typical features to face-to-face communication, considered the most natural form of communication. According to MNT, a decrease in medium naturalness results in an increased cognitive load imposed on the learner, extra ambiguity in the message conveyed, and lower psychological arousal during the interaction, and all these factors negatively affect learning effectiveness (Kock, 2005; Kock et al., 2007; Weiser et al., 2018).

MNT describes five criteria for evaluating the degree of naturalness of a communication channel: (1) physical colocation, (2) level of synchronicity, (3) identification and transmission of facial expressions, (4) identification and transmission of body language, and (5) receiving and transmitting natural speech. Due to the inherent limitations of online learning, the first criterion is clearly not feasible. In synchronous online lessons, the second criterion is provided and the third and fifth criteria are also commonly met. As detailed above, the fourth criterion is usually only partially met, as often only the faces of the participants are displayed.

The developed tool primarily addresses the first and fourth criteria. Although the first criterion usually refers to the fact that the instructor and students are co-located in a common physical space, we extend this criterion with a third element: the content. In traditional classrooms, usually, a scene comes to life in front of the students in which the instructor and the content are present together. As the instructor explains in front of the projected slides (or blackboard), they sometimes “move closer to the content”, point to key elements, etc. In synchronous online learning environments, the virtual colocation is weaker, usually consisting of a small overlay (so the background of the teacher is not distracting) of the instructor on the slides. Our goal was to improve on this by removing the clutter (background), extracting only the instructor, and prominently projecting their figure onto the slides, hopefully bringing what appears in front of online students closer to their face-to-face experience.

Moreover, the new tool offers an extra layer of interactivity, as the teacher has real-time feedback on its placement relative to the content, enabling him/her to freely move between the elements of the slide. Although the instructor’s body also appears on the slides in a number of asynchronous videos, since these scenes were prerecorded, they lack a live instructor-content communication. Interestingly, Aydin et al. (2013) and Valdivieso et al. (2013) have remarked that the teacher’s ability to improvise and adapt to the live environment and dynamics strengthens the classroom bond and has a large positive impact on work performance and the teaching-learning process.

**Fig. 2:**
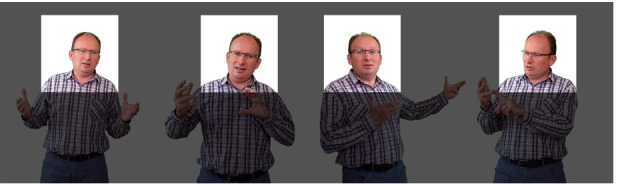
Body language is lost in the typical laptop webcamera feeds

Regarding the fourth criterion of MNT, limiting the instructor’s body language inevitably contributes to a decrease in instructor expressiveness. Previous research confirmed the importance of instructor expressiveness in promoting effective learning. For example, Wang et al. (2019) found that an increase in instructor’s facial expressiveness resulted in increased student arousal level and learning satisfaction. Another way to increase the expressiveness of online lectures is to exhibit the full spectrum of the instructor’s body language, showing much more than a face. As shown in Fig. 2, in a typical online lecture streaming setting, where the teacher is seated in front of a laptop, most of the body language cannot be perceived by listeners. We approached the problem from this perspective.

### Emotional body language

Besides the CoI and MNT perspectives described above, it is worth examining the importance of body language in communication through the prism of current findings in neuroscience and psychological sciences. After noticing that in the past researchers in Affective Neuroscience have focused mainly on studying the importance of emotional facial expressions, Calbi et al. (2017) state that recent results emphasize the fundamental role emotional body language plays during social interactions. For example, research has shown that the perception of facial expressions is influenced by bodily expressions (Meeren et al., 2005; Van den Stock & de Gelder, 2014). Aviezer et al. (2012) report on the so-called “composite person effect” and emphasize that faces and bodies are processed as a unit. In line with this, Van den Stock et al. (2007) conclude that their findings show the significance of “emotional whole-body expression” in communication. In addition, De Gelder et al. (2010) accentuate that, although both the face and the body convey information that is essential for social interaction, they each fulfill this role differently. Although all these findings underline the relevance of body language (gestures, posture, etc.) in expressing our emotional and mental states, its contribution, for example, to instructor social presence is an underresearched area.

Another important finding regarding the importance of emotional body language is that nonverbal communication also affects the individual. Recently, Puertas-Molero et al. (2018) reported on a large-scale research that aimed to reveal potential connections, relations and associations among perceptions of stress, burnout syndrome, emotional intelligence, and nonverbal communication.

## Research methodology

### A client-side web application for synchronous online presentations

The developed application segments the presenter’s body (removing the background) from the webcamera’s video feed and projects it onto a set of slides. It runs entirely locally in the web browser; therefore, it is platform independent, there is no installation required, and it ensures maximal privacy.

**Fig. 3:**
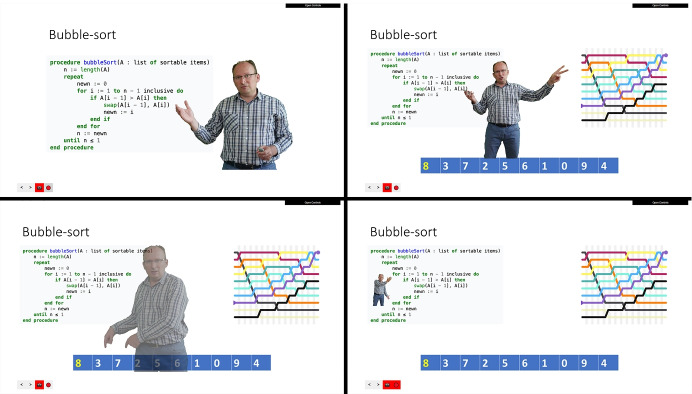
Broad on-slide presence: the position, display size of the instructor can be dynamically customized with the help of a wireless presenter, equipped with a built-in touchpad and fine-grained control and pointing capabilities

The presentation can be controlled with a wireless presenter, allowing the instructor to easily and quickly navigate the content, engage a virtual laser pointer, toggle the camera and background, and reposition and resize itself on the slides as exemplified in Fig. 3.

The application is freely available for anyone to use at the following address: https://www.ms.sapientia.ro/~iclanzan/prezcam/.

### Research context

The current research includes four studies conducted at the Sapientia Hungarian University of Transylvania, Faculty of Technical and Human Sciences Târgu-Mureş, and aims to explore whether projecting the instructor’s body onto the slides (“on-slide presence” format) improves IP. We have proposed to test the effectiveness of this learning format relative to the settings of “just narration” and “image off-slide” (instructor’s face next to the slides) in terms of subjective evaluation of the learning process by the learners (pilot study and Study 2) and the choice of the students (Study 3). We were also interested in teacher feedback on the potential benefits and disadvantages of the proposed system (Study 4).

Previous studies investigated mainly the effect of incorporating the instructor’s face in slide-based video lectures and revealed that students prefer this format (relative to the “just narration” format) for the self-reported reason that this presentation style supported them in being more focused and feeling more connected (Kizilcec et al., 2015). The findings in this field also support the image principle of multimedia learning that states that students do not necessarily learn better from a multimedia lesson when the instructor’s image is added to the screen (Homer et al., 2008; Kizilcec et al., 2015; Wilson et al., 2018). Two possible reasons have been suggested: i) the resulting social cue is too weak to induce such positive social responses in learners, which could surpass the generated attention division between the two video inputs (instructor versus slides) (Kizilcec et al., 2014); ii) the instructor’s body language (gaze guidance, hand gestures, body posture, etc.) is not available to guide the student’s attentional focus or to accentuate key aspects of the presentation (Stull et al., 2018). Despite this, Wilson et al. (2018) underline the importance of taking subjective feedback from students into account, as these evaluations are likely to guide the decisions students make about determining the learning resources they use, affecting their learning outcomes. Kizilcec et al. (2015) also concluded that although social and other nonverbal cues may not enhance learning per se, these factors have the potential to increase student motivation to persist in an online course, which is expected to benefit them in the long run.

Based on these findings, we anticipated that in synchronous learning environments, students will prefer the “image off-slide” format over the “just narration”.

The above detailed literature review on CoI, MNT, and Affective Neuroscience perspectives on the importance of body language in communication suggests that projecting the instructor’s body onto the slides may contribute more strongly to IP than the “image off-slide” format (talking head next to the slides). Consequently, we hypothesized students will rank the three examined synchronous online learning formats in the following order: “just narration” (less preferred), “image off-slide”, “on-slide presence” (most preferred).

#### Pilot study

After the first version of the application had been developed, we conducted a pilot study (Iclanzan & Katai, 2021) to test the effectiveness of the generated learning environment. The system did not yet include the wireless presenter, and we used a digital green screen. Four slide-based online lectures were included in this study, attended by 134 first and second year students from 10 different study programs. During the previous 13 weeks of the semester, participants attended these courses in the “image off-slide” format. As for the other courses taken by the participants, most were also slide-based, but held in the “just narration” format. The questionnaire administered to the participants after the lecture asked them to rank the three experienced lecture formats. Contrary to our expectations, the results revealed two major camps at the opposite ends of the IP spectrum. The largest, with 43.28% of the first choices, was the “on-slide presence” presentation approach. However, almost as many students designated (41.29%) as their first choice, the “just narration” option. The vast majority of the respondents (73.88%) stipulated as their second choice the “image off-slide” approach. Therefore, this option seems to offer a suitable compromise for the two preference groups.

**Fig. 4:**
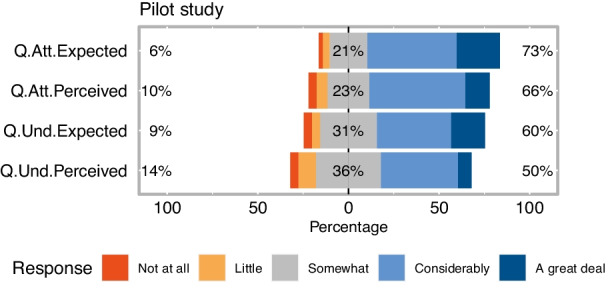
General expectations and perceived benefits after a one-week pilot run of the “teacher-overlaid” format

We also asked subjects to evaluate the importance of the instructor’s body language to engage attention and facilitate better understanding. We were curious about their opinion in general, but also about what they experienced with the new lecture format. Figure 4 presents the Likert plots of the responses.

Most students considered that a stronger visual presence of the teacher would help them to be more focused during the online lectures and would be helpful in fostering a better understanding. Although their self-assessed scores regarding the new delivery method were also high, these values were much lower than with general opinion items. Differences are statistically significant (*p* < 0.001 in both cases), as per the modified Wilcoxon signed-rank test proposed by Derrick & White (2017) for highly correlated paired data. This result made us aware that students anticipate more potential in nonverbal communication elements than we could exploit in the context of the pilot study.

### Study 2

#### Research questions and hypotheses

Based on the student feedback from the pilot study, we improved the tool and adjusted teaching strategies, increasing instructor-content interaction, and minimizing content obstruction. During the second semester, one course was implemented almost entirely using the updated version of the application. In the last two weeks, we switched to the other two formats. This enabled students to experience all three delivery formats in the same course and had a fresh take before the second measurement, which focused on the subjective evaluation of the learning process by students. We were interested in whether they appreciated mainly the new format because of its novelty or whether students will find it useful even after a one-semester use. We expected the participants to rank the three delivery methods in the same order as they did in the pilot study (Hypothesis 1): “image off-slides” (less preferred), “just narration”, “on-slide presence” (most preferred).

Considering the above detailed facets of the construct of IP, students’ self-assessment questionnaire included five components. As in the case of the pilot study, the first three components were: student satisfaction and preferences (factor 1), perceived attentional engagement (factor 2), and perceived learning (factor 3). Given the third subdimension of the TP component, we expected an increase in IP to be reflected in students’ perceived attentional engagement and level of understanding of the presented subject. One of the factors through which students can perceive the level at which the second subdimension of TP is manifested is the conversational style (factor 4) of the lecture (Dudley-Evans, 1994). Martin et al. (2018) highlight that teaching presence occurs, for example, when the instructor interacts with students, encouraging them to actively participate in the course. These authors also emphasize the importance of instructor connectedness, which can be defined as the “perceived closeness between the student and instructor as well as the instructor and student” (D’Alba, 2014, p. 8) According to Lujan and DiCarlo (2017), establishing a personal connection will result in more inspired and engaged students. Due to its SP component, it is likely that an increase in IP will result in students perceiving the lecture as more personal (factor 5). Consequently, the hypotheses tested relate to how the examined teaching-learning format contributes to:

- (H2.1) making the lecture more enjoyable (student satisfaction);
- (H2.2) students’ perceived attentional engagement;
- (H2.3) students’ perceived level of understanding;
- (H2.4) make the lecture more conversational;
- (H2.5) make the lecture more personal.

#### Participants

Of the 55 course attendants, 48 answered an anonymous questionnaire and provided feedback on their experiences and impressions. 12 (25%) of the responders were females and 36 (75%) males. Most students were freshmen studying Informatics (29 (50.4%)), and the rest, 18 (37.5%) sophomores and 1 junior participant, were enrolled in the Computer Science study program.

#### Procedures and measurements

We asked respondents to complete a brief anonymous Google Forms survey. After supplying some demographic information regarding their major and sex, the students provided feedback regarding the

- online lectures delivered with the developed system, and
- perceived self-reported impact (if any) along different dimensions.

The survey instructed respondents to reflect on the lectures delivered in the new format throughout the semester and compare their impact relative to lectures delivered in the other formats (factor 1). Then, they were asked to assess how much the “on-slide presence” lecture format helped them (if at all) regarding factors 2-5 discussed in Section 3.3.1. The responses were recorded on a five-level unipolar Likert scale ranging from “not at all” to “a great deal”, quantifying perceived benefits (if any). The structure of the survey and how the questions relate to the established factors and hypotheses are described in Table 1.

**Table 1:** Structure of the survey completed by the students at the end of the course

Hypothesis	Question Name	Item
1. H2.1 (Students’ satisfaction and preferences)	Q.More	Yes-or-no question, whether they would like to attend online lectures delivered by the teacher overlaid on the slides format.
	Q.Overall	Overall impression of the course format on a five-level Likert scale, from “very disturbing” to “very useful”.
	Q.Disturbance	Feedback on how often the projection of the instructor onto the slides was distracting, disrupting, etc., on a five-level Likert scale, from “very often” to “never”.
	Q.Format Preferences	Pairwise comparison, favorite selection of the three experienced online teaching formats.
2. H2.2	Q.Attention	Attentional engagement.
3. H2.3	Q.Understanding	Level of understanding of the subjects presented during the lecture.
4. H2.4	Q.Dialogue	Level of meaningful interaction and conversation.
5. H2.5	Q.Personal	Instructor connectedness, sense of community, and belongingness.

At the end of the survey, in the two free text fields, respondents could describe in which circumstances they experienced distractions or disruptions induced by the new lecture format and provide general feedback and suggestions for improvement, sharing their thoughts regarding the experienced lecture format.

#### Results

**Fig. 5:**
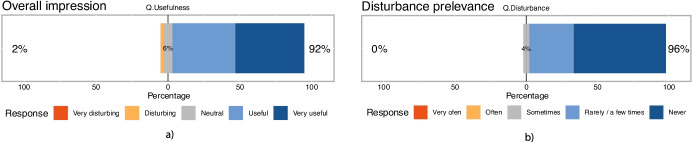
Evaluation of **a**) overall impression and **b**) frequency of inconveniences, disturbances resulting from the “on-slide presence” format

46 students reported they would like to attend future lectures in the new format (*Q.More*), and two were opposed to the idea. The distributions of responses for *Q.Overall* and *Q.Disturbance* are presented in Fig. 5. The responses are heavily right skewed, with 91.6% indicating that the “on-slide presence” format in synchronous online learning is “very useful” or “useful” and 95.83% reported that they were “never” or “rarely” distracted or annoyed by the teacher’s depiction on the slides. One student considered that the new format is overall “disturbing” and three others marked “neutral” in their responses .

**Fig. 6:**
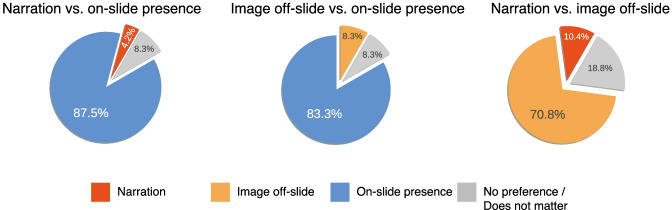
Students’ preference on synchronous online teaching formats

The outcome of the pairwise comparison between the three formats (*Q.Format Preferences*) is shown in Fig. 6. Three students did not see personally meaningful differences between the formats and chose “no preference” for all three comparisons. The “on-slide presence” was by far the first ranking format. Six students (12.5%) had a preference against this presentation style; when given the option, four of them chose the “image off-slide”, and two the “just narration” format.

**Fig. 7:**
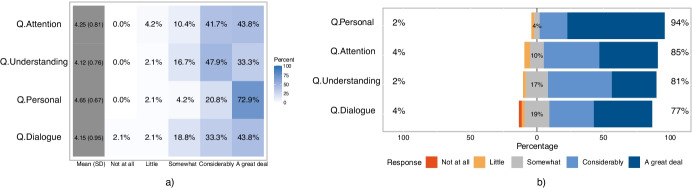
Evaluation of the self-reported impact of the “on-slide presence” format across various dimensions: **a**) heatmap table representation; **b**) likert plot

Figure 7 presents the results for the perceived impact of the new synchronous online lecture format along factors 2-5. On the left, in Subfigure a), we present a heat map of the response distribution for each dimension (rounded to the first decimal place), in a separate row. The first (grey) column contains the mean of the scores from the Likert scale (from 1 - “not at all” to 5 - “a great deal”) and their standard deviation (SD). On the right, Subfigure b) depicts the Likert plots for each component, ranked by the magnitude of the self-reported impact.

Across all dimensions, we had a single instance of a “not at all” choice, for *Q.Dialogue*. All other responses indicate at least some, and in the majority of cases a very significant impact. Consequently, the mean scores of the perceived benefits are very high, exceeding 4.1 for each component.

The greatest effect was reported for *Q.Personal*, 93.75% of the responses considered that the lecture format helped “a great deal” or “considerably” to achieve more personal lectures, with greater instructor connectedness.

Similar to the pilot study, the very strong perceived beneficial effect for attention exceeds that for understanding (85.41% vs. 81.25% ). However, contrary to our first experiment, after almost a semester of teaching with the improved tool, the perceived impact of both aspects exceeds the expectations and general opinion of the students, regarding how much an increased teacher presence could benefit them. We evaluated the general opinion on a much larger body of students from various study programs, while the second study concerned only students from two majors. To eliminate a possible bias, we also evaluated the general opinion of the pilot study only for students majoring in the same two fields, namely, Informatics (I.) and Computer Science (CS.) (72 cases out of 134).

On that account, the first two columns of Table 2 contain the mean scores and standard deviations of the expected benefits for attention and understanding, as reported in the pilot study by students from all study programs, then only the ones majoring in I. or CS. To facilitate a quick comparison at a glance, the third column again contains the mean scores and standard deviations for the perceived benefits collected from the students involved in study 2 (also presented in Fig. 7a)). Indeed, the mean scores for the expected benefits are much lower, well below 4. We used the Mann-Whitney U-test to confirm that the differences between the expected and perceived benefit scores are statistically significant (*p* < 0.01 for all cases).

**Table 2:** Mean scores and their standard deviation for the expected benefits (from the pilot study) and the perceived benefits reported in study 2

Component	Pilot study - General opinion, benefit expectations		Study 2 - Perceived benefits
	All study programs	Just I. and CS.	I. and CS.
Attention	3.89 (0.89)	3.74 (0.92)	4.25 (0.81)
Understanding	3.65 (0.98)	3.42 (0.99)	4.12 (0.76)

We also calculated the strength of the improvement over the expectations using the Common Language Effect Size (CLES) (McGraw & Wong, 1992) and Cohen’s *d* (Cohen, 1988). The effect sizes are presented in Table 3. The CLES values are straightforward to interpret, as they present the probability of superiority of the perceived benefits over the expected ones. Depending on the component and comparison group, the probability of a higher perceived score is in the 62-70% interval. Cohen’s *d* scores also reveal that the effect sizes are mostly medium (0.5 threshold) and toward large (0.8 threshold) in the case of understanding in the I. and CS. comparison group.

**Table 3:** Evaluation of the strength of the differences between the perceived benefit scores and the expected ones. Common Language Effect Size (CLES) and Cohen’s *d*

Component	CLES (*d*_*Cohen*_) perceived vs. expected	
	All study programs	Just I. and CS.
Attention	61.86% (0.416)	66.22% (0.585)
Understanding	63.61% (0.511)	70.08% (0.782)

Table 4 enumerates the choice distribution for the high (“great deal” or “considerably”) expected benefits from the pilot study, and the perceived high benefits from study 2. As seen in the two rows labeled “Total”, the differences when compared to the corresponding I. and CS. groups are even higher: 66.66% vs. 85.41% for attention, 54.16% vs. 81.24% for understanding. The increases are especially stark at the right end of the Likert scales. Only 18.05% and 8.33% of I. and CS. students had the expectation that an increased instructor presence in online lectures could help them a “great deal” in paying attention and in better understanding of the material. However, the same magnitude of actual perceived benefits were reported by 43.73% and 33.33% of the respondents, almost two and a half and a four-fold growth.

**Table 4:** Comparison between self-reported benefit of students (from the pilot study) and perceived benefits reported in Study 2

Component	Impact degree	Pilot study - Benefit expectations		Study 2 - Perceived benefits
		All study programs	Just I. and CS.	I. and CS.
Attention	Great deal	23.88%	18.05%	43.75%
	Considerably	49.25%	48.61%	41.66%
	**Total**	**73.13%**	**66.66%**	**85.41%**
Understanding	Great deal	18.65%	8.33%	33.33%
	Considerably	41.04%	45.83%	47.91%
	**Total**	**59.69%**	**54.16%**	**81.24%**

For an instant overview, the totals are represented in bold

In Fig. 7b) we see that the *Q.Dialogue* component has the weakest impact. 77.08% of the respondents considered that the teaching format had considerably or greatly facilitated interaction and conversation. One student did not perceive any benefit along this aspect.

Students provided 14 entries in the feedback-suggestions field of the survey. All of them had a positive tone and the two most common themes revolved around the importance of instructor gesturing and signaling and the novel and more personal nature of the lectures. One student expressed the view that the teacher’s personality heavily influences the success of this presentation format and is mainly suited for instructors who “explain enthusiastically”, with a lively body language.

#### Structural equation modelling

As shown in Fig. 1, the CoI framework defines at the intersections of the presences three sub-elements: “Supporting Discourse” (SD), “Setting Climate” (SC) and “Regulating Learning” (RL). To better understand the interplay and influence of the factors along the social and cognitive dimensions on the educational experience, we also built and analyzed a structural model, using the questionnaire answers as indicators to the aforementioned three constructs. While the questionnaire was designed to be very brief, each sub-element was partially evaluated by at least one survey item. SD is reflected in *Q.Dialogue*, SC in *Q.Personal*, RL in *Q.Attention* and *Q.Understanding*, while the “Satisfaction with the Educational Experience” (EE) is measured by *Q.More, Q.Overall, Q.Disturbance* and the choices regarding the on-slide format expressed in the *Q.FormatPreference* pairwise comparisons.

Due to the low sample size (Reinartz et al., 2009), having single-item variables (Garson, 2016, p. 31), and all factors being reflective, we fitted the model using the consistent Partial Least Square Structural Equation Modelling (PLSc-SEM) (Dijkstra & Henseler, 2015) algorithm. The method analyzes and redresses the correlations of reflective constructs, making the results consistent with a factor-model.

The constructs were subjected to confirmatory factor analysis to test if the data fits the measurement and structural model. Due to their low variance, *Q.More* and *Q.Disturbance* did not load well, having outer loading scores well bellow the 0.5 cutoff, and consequently were removed from the model.

The reliability of the constructs with multiple indicators was measured using Cronbach Alpha and Composite Reliability. The obtained scores, 0.827 / 0.832 for RL and 0.790 / 0.804 for EE indicate that a good reliability is attained (scores > 0.7). The Average Variance Extracted was above 0.5 for all constructs, showing also a proper validity.

The discriminant validity of the concepts was assessed using the Heterotrait-Monotrait (HTMT) ratio of correlations. All ratios were less than the 0.9 threshold, ranging from 0.154 in the case of SC - RL to a quite high value of 0.862 for RL - EE.

The statistical significance of the various PLSc-SEM results was assessed by using consistent PLS bootstrapping with 10000 subsamples.

**Fig. 8:**
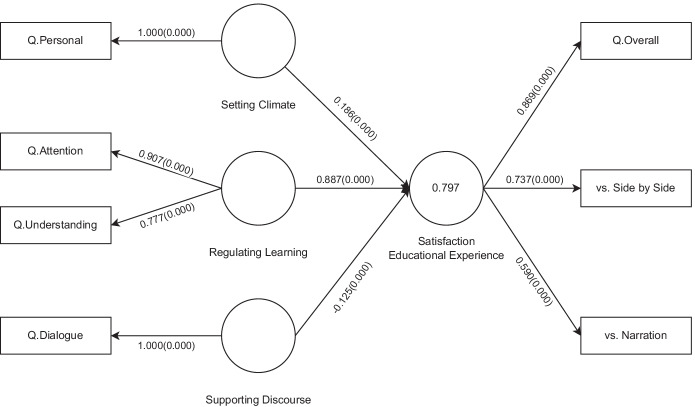
PLS path model and estimation of results

Figure 8 presents the analyzed model (adequate fit, SRMR= 0.073), with *p* values in parentheses. As already expected due to the high HTMT ratio close to the 0.9 limit, the association between RL and EE is the strongest, accounting for the vast majority of variance in EE. This stems from the strong correlation of *Q.Attention* with both *Q.Overall* and format preferences. Namely, those who reported the highest perceived attention benefit (*Q.Attention* = 5) always chose the on-slide format and had a very high average *Q.Overall* score of 4.8095 out of 5.

The analysis also uncovered a smaller magnitude negative relationship between SD and EE. Looking at the responses we saw a reversing trend of *Q.Dialogue* as a function of overall satisfaction. Those who were not very satisfied with the format (*Q.Overall*≤ 3) tended to score *Q.Dialogue* > 3 ≥ *Q.Overall*, even indicating a score of 5 in 40% of cases. However, those who were very satisfied (*Q.Overall*= 5), tended to score *Q.Dialogue* < *Q.Overall*, 4.26 on average. This result again emphasizes the major role of perceived learning benefits in student contentment. Other factors by their own do not raise the satisfaction to match their levels.

### Study 3

#### Research questions and hypotheses

To not only rely on students’ self-assessment and eliminate the possibility of conformity, leniency, and rater biases, we designed a third experiment where students exercise their own independent judgment, and we analyze the distribution of their uninfluenced choices. The study overlapped with the last part of study 2. We were interested in whether the actual behavior of the students would be in line with their perception (Bandura, 1986) (whether the results of the third study confirm the results of the second one). We focused on the “just narration” vs. “on-slide presence” comparison, since participants in the pilot study appreciated these formats the most. We hypothesized that if participants found the new delivery method to be more effective and engaging, they would prefer, and given the option, choose this lecture format (Hypothesis 3).

#### Participants, procedure and measurement

The study involved two teachers and other student participants. Neither the teachers nor the students involved were familiar with the tool or the research hypotheses behind the study.

The third study included two one-hour presentations, where the first 15 minutes of the lecture in “just narration” format were followed by 15 minutes of “on-slide presence” format (or conversely). Then, after a brief break, the participants choose in which format they wish to continue the lecture. For the first online lecture, covering an interdisciplinary topic regarding art and computing, we had 25 participants. 75 students attended the second lecture, where real-world applications of partial differential equations were discussed. Before the lectures, the teachers were shown how to use the tool and the presentation remote.

#### Results

In the case of the first lecture, only 1 out of 25 students opted for the “just narration” format. For the second, more technical topic, 9 out of 75 (12%) students choose this format, focused more on content.

### Study 4

Finally, in the fourth study, we interviewed four colleagues who had used the new tool to implement at least one lecture. Besides focusing on the above aspects from the instructor’s perspective, the interviews also addressed the impact of the generated teaching experience on the instructor.

#### Participants and data collection

We interviewed the subjects using contextual interviews (Saldaña, 2021). The semi-structured interviews were conducted (within 48 hours following the final teaching experience with the new format) with four university teachers: two assistant professors and two assistant lecturers. The interviews were conducted through the Google Meet platform with cameras turned on. During the 50- to 70-minute interviews, teachers reflected on three topics: i) How they experienced the transition to online education, what were the challenges, and what impact did it have on the educational process (both from the perspective of the teacher and the students)? ii) From a year’s perspective, how do they look back at that period? iii) What are their impressions, comments, suggestions, etc. regarding the tool used and the generated “on-slide presence” format? We explicitly asked teachers to rank the three examined lecture formats and to anticipate the ranking of the students. The interview script was adjusted to the line of discussion. Following the transcription of the recorded interviews, the data was coded and analyzed manually.

#### Data analysis

Our qualitative data analysis used first cycle coding methods - descriptive evaluation and structural coding - as described in Saldaña (2021). At the first level, we defined two code folders: “perceived student benefits” and “impact on the instructor”. Subcodes were created within both categories. These subcodes addressed the same components that we included in our Study 2 analysis. Subcodes “student satisfactiont”, “attentional engagement”, and “level of understanding” (as the instructor perceived the extent of these factors) were attached to the first code folder. In the second code folder, the following subcodes were included: “conversational style”, “instructor connectedness”, and “instructor content interaction”. Since we were interested in the impact of the new format on the instructor, we also added the “instructor satisfaction” subcode to the second folder. Besides these predefined codes, a subcode “expression of personal identity” was subsequently added to folder 2. During the data analysis, we paid special attention to contrasts between the experiences generated by the previously used formats and the new format (for example, online fatigue and burnout syndrome versus student and teacher satisfaction).

#### Findings

Regarding teachers’ general impressions on the transition to online teaching, all narratives revealed positive first reactions which were later transformed into negative feelings due to general student passivity, online fatigue and burnout for both teachers and students. The subjects uniformly emphasized the lack of personal relationships and personal presence. Two of them stressed the acceleration of sedentarism and the associated health toll for teachers.

Concerning the new format, teachers commonly evoked three types of benefits. Interpersonal (social) benefits are the closer teacher-student relationship, the social belonging effect, the fact that the personality of the teacher is present in the class, and this allows for the building of trust between parties. The health benefits are linked to the reduction of sedentarism by standing up and moving. This body position also allows a broader range of exposition possibilities through face gestures and body language. Third, there are substantial technical advantages recalled: the software allows for simultaneous teacher presence and material presentation, the real-to-life situation resembles being personally in the class, and the dynamism and interaction help maintain attention for a longer time.

Three of the interviewed teachers emphasized that the software was motivating, as it required them to prepare, get dressed properly, and physically prepare for classes. Two teachers noticed that in the lack of feedback from the students, the program itself provides some feedback to the teacher. The possibility to see themselves and not only the screen while speaking confers self-confidence.

## Discussion

The four studies in tandem support the adequacy of the studied teaching-learning format for synchronous online lectures and the research hypotheses derived from the related literature review.

The results of Study 2 on the item *Q.FormatPreferences* confirmed our first hypothesis in the sense that students prefer the new format (“on-slide presence”) over the other two formats examined (“just narration”, “image off-slide”). The vast majority of participants chose this format in both the “on-slide presence” vs. “just narration” and the “on-slide presence” vs. “image off-slide” comparisons. One possible explanation for the final placement of the “just narration” format is that after the students had been accustomed to seeing the teacher for weeks, they became averse to this format. Considering the students’ responses to *Q.More*, *Q.Overall*, *Q.Disturbance*, and *Q.FormatPreferences* as complementary aspects of expressed student satisfaction, we can state that the results also support H2.1, that is, projecting the instructor’s appearance onto the slides contributes to making the lecture more enjoyable. The results of Study 3 reinforced these conclusions in terms of actual behavior and confirmed H3.

Although the order set up by the students (after using the new tool for almost one semester) in terms of the three formats differs from that of the one-week pilot study suggested, it is in line with related previous research in the field of asynchronous online learning. For example, Kizilcec et al. (2015) compared two learning formats (during a 10-week course) that included lecture videos with and without the instructor’s face. These authors found that most learners preferred to watch lectures with the instructor’s face (talking head in the lower right corner of the video) for the self-reported reason that social and other nonverbal cues from the instructor helped them to be more focused and feel more connected. More recently, Wang et al. (2020) also reported that integrating a real instructor on the video screen positively affected students’ satisfaction, and situational interest. In addition, Wang et al. (2019) found that students preferred video lectures with a heightened level of instructor expressiveness much better than those with a conventional level of expressiveness or audio-only ones. Consequently, our finding that exhibiting the full spectrum of instructor body language (by projecting it onto slides) results in an additional increase in student satisfaction is a natural extension of previous results.

The results based on the students’ answers to the questions Q.Attention, Q.Understanding, Q.Personal, and Q.Dialogue confirmed hypotheses 2.2-5 and provide further insight into why students rated the new format so highly. The effect is reported as considerable or greater by the vast majority of students. In the case of the attention and understanding components, the reported benefits significantly exceeded even the expectations measured in the pilot study. The measured effect sizes are medium to large when compared to these expectations. The growth along the understanding component is particularly prominent.

The structural equation modelling also revealed the importance of perceived learning benefits in student satisfaction. Especially those who can pay better attention than otherwise, enjoy and prefer the new format. This is in line with the conclusion reached by Ghaderizefreh and Hoover (2018), that higher level of understandability and fostering attention contribute to increased students satisfaction with the online learning experience.

Wang et al. (2020) observed a similar phenomenon in the case of video lectures presenting difficult topics. One of the conclusions of this study was that perceiving the instructors’ social cues may facilitate the processing of conceptually relevant information and this gain may outweigh the expense of divided attention between the two video inputs.

A further plausible explanation for our result might be that the new format enables a more effective teacher-content interaction and more engaging teacher-student interaction contributing significantly to the TP component of CoI (2nd and 3rd subdimensions). The initiators of the CoI model have emphasized from the beginning that TP has a binding effect in the creation of CoI (Garrison et al., 1999) and serves as the major influence that sets the tone for the overall learning experience (Garrison et al., 2010). Recently, Çakıroğlu & Kılıç (2020) confirmed the key role of TP within the framework of synchronous online learning. With regard to the remote participants in a blended synchronous learning environment, Shi et al. (2021) also found that increasing the level of perceived pedagogical affordance would result in increased learning motivation and stimulate students to adopt meaningful processing strategies.

The two new components included in Study 2 related to interpersonal relationships (items *Q.Dialogue* and *Q.Personal*). *Q.Dialogue* scores attest that the proposed format can help the instructor engage learners in content-related discussions more efficiently. The responses of the students supported H2.5 the most. The high *Q.Personal* scores are in line with those studies that conclude that measures of lecturer personality traits (Patrick, 2011) correlate with measures of student course satisfaction. Other studies reported that the instructor’s manner of presentation also influences student engagement with their studies (Borup et al., 2011; Ladyshewsky, 2013). Since human personalities are reflected in their whole being and the teacher’s manner of presentation most often involves the entire spectrum of their body language, it is not surprising that the participants perceived such a significant contribution to this factor.

The Crook & Schofield (2017) study provides further explanation why the format we examined proved to be so appreciated. Three key elements of it were: synchronicity, perceptibility of the full body language of the instructor, and facilitating instructor-content interaction. All these components have the potential to contribute to the majority of the key features that Crook and Schofield (2017) have identified as characterizing captivating traditional face-to-face lectures: intersubjectivity, agency, embodiment, expression of personal identity, and lecture as episode.

For example, the management of intersubjectivity is defined as animating an implicit dialogue with the silent audience and is related to the concept of instructor immediacy’ in the live classroom (Mehrabian, 1971). This management strongly depends on how the demeanor, facial expression, and body language of the instructor are integrated with speech (Crook & Schofield, 2017). The construct of agency clearly assumes a level of synchronous social interaction that is implicit in synchronous learning formats. Considering lecturing as an “embodied” form of activity again underlines the importance of those communicative actions that are concentrated on the body and its posture, movements, and gestures. As (Hwang & Roth, 2011, p. 464) noted: “students do not perceive what might be in the head of the lecturer—what they concretely perceive is his/her vocal, gestural, and positioned bodily performance of concepts in the here and now of the classroom”. Lecturing can be seen as an act of personal exposure in the sense that it reveals how a particular instructor personally relates to the taught material (Goffman, 1981). By projecting its appearance onto the slides, we give the instructor a similar opportunity as in face-to-face education to convey its identity more accurately, realistically, and authentically (Baran et al., 2013)

Teacher interviews supported most of our student perspective hypotheses and revealed some other important aspects. All teachers appreciated primarily the “on-slide presence” format and expected to be the first choice of students as well (H1 and H3). Common elements in the interviews were that the proposed format supported teachers in explaining slide content better (H2.3) and the increased dynamism and teacher-content interaction resulted in increased attentional engagement (H2.1). Regarding the social benefits, all teachers emphasized that the new format contributed to online lectures becoming more personal and human centered (H2.5). On the other hand, none of the teachers mentioned that the students had become more active, for example, by asking more questions. Interestingly, the students’ scores were lowest for H2.4 (item *Q.Dialogue*). This aspect draws attention to the fact that the “on-slide presence” format does not implicitly train students to ask more questions. Martin et al. (2018) also emphasized that students need explicit stimuli to actively participate in the course.

In addition to these aspects, the interview answers revealed that the teachers were very excited about their teaching experience with the new tool and format. They stressed that it helped them express their personal identity more authentically. Some comments in this sense are: “It helped me better showcase my teaching expressiveness”; “I felt much better; and if the teacher feels better, it has an impact on the quality of its lessons”; “I hope students will now recognize me on the street”; “It was like a wake-up call, revealing my uncomfortable comfort zone”. Puertas-Molero et al. (2018) also found that both emotional clarity and emotional regulation relate positively with the use of body language, and emotional intelligence and body language are two significant factors in the prevention of burnout syndrome.

### Limitations

Although the results of the studies are promising, there are some limitations along with opportunities for future research directions.

First, only one longer-term study (one semester) was conducted. It is not yet clear how the results generalize to other teachers and materials. Instructors’ personality and teaching style might heavily influence the perceived benefits. A larger-scale study would be needed to determine whether, in general, there are significant differences in outcomes and student satisfaction between the different synchronous online teaching formats.

Furthermore, the students experienced only one course delivered in the “on-slide presence” format. Although many of the reported benefits could indeed be due to the inherent value of the format, some might stem from the current uniqueness, novel format of the course. Again, further longitudinal studies are necessary to determine how the benefits hold up once the format becomes more prevalent and the novelty factor wears off.

By deliberately designing the survey short, the mensuration of the intersecting components in the CoI framework was only partially covered, and the analyzed structural model contains single indicator latent variables. A more detailed questionnaire, covering distinct facets such as immediacy, intimacy, humanness, expression of personality, clarity of design, pace, interaction, integration of discourse and reflection etc. is needed to gain a better insight in these complex overlapping components, and their relationship with the educational experience.

## Conclusions

To help mitigate the effects of online fatigue and isolation, and to improve the overall online learning experience of students, we developed a freely available and easy-to-use web application that projects in real time the teacher’s figure on the slides. We conducted a pilot study and later two other studies, based on the subjective evaluation of the students, to examine the quality of the learning experience generated. A fourth investigation focused on the instructor’s perspective.

The results revealed that the lectures supported by the new tool had an increased instructor presence with a very meaningful impact on student satisfaction and other four related components. Significant positive effects were measured for perceived learning and attentional engagement. According to the students’ concordant feedback, lectures became far more personal with increased teacher connectedness.

The experience accumulated with the longer-term use and the gathered feedback also revealed the importance of adequate instructional strategies. The teacher’s placement on the slides is paramount and might require careful forethoughtful planning.

Teacher-content interaction and lively body language are beneficial and appreciated, while obscuring of content and frequent repositioning are disturbing and should be avoided.

Based on these findings, where appropriate, we recommend using the “on-slide presence” synchronous online delivery format, as it benefits the learning process, increases student satisfaction and teacher self-awareness, and can reduce online fatigue for both categories of participants.

## Data Availability

The datasets generated during and analysed during the current study are available from the corresponding author on reasonable request.
